# Health team practices to improve vaccination coverage of children in a *favela*

**DOI:** 10.1590/1980-220X-REEUSP-2024-0337en

**Published:** 2025-06-06

**Authors:** Gean Mascaranhas Gomes, Giulia Gazineo Trindade Assis, Sandra Cristina de Souza Borges Silva, Liana Amorim Corrêa Trotte, Marluci Andrade Conceição Stipp

**Affiliations:** 1 Universidade Federal do Rio de Janeiro, Centro de Ciências da Saúde, Escola de Enfermagem Anna Nery, Rio de Janeiro, RJ, Brazil.; 2 Universidade do Estado do Rio de Janeiro, Faculdade de Enfermagem, Departamento de Enfermagem Materno-Infantil, Rio de Janeiro, RJ, Brazil.

**Keywords:** Vaccination Coverage, Child Health, Health Management, Primary Health Care, Nursing

## Abstract

**Introduction:**

The Brazilian National Immunization Program currently faces numerous challenges, such as vaccine hesitancy, the spread of fake news, and the impacts of COVID-19, especially in vulnerable communities, where social and structural barriers affect vaccination coverage.

**Objective:**

To report the experience of planning and implementing practices to expand vaccination coverage for children up to two years old in a favela in Rio de Janeiro.

**Method:**

An experience report carried out between June and November 2023, structured in two stages: planning, using GeoVacina Rio to identify vaccination gaps; and execution, which included active search, vaccination “D-Days”, educational activities and digital communication with guardians.

**Results:**

100% vaccination coverage was achieved for registered children, strengthening ties with the community and reducing access barriers through integrated and humanized strategies.

**Conclusion:**

The use of technology, health education and humanized care proved effective in expanding vaccination coverage in vulnerable areas, serving as a model for other locations.

## INTRODUCTION

The Brazilian National Immunization Program (In Portuguese, *Programa Nacional de Imunizações* - PNI), coordinated by the Ministry of Health, with actions shared with state and municipal health departments, completed 51 years of its history in 2024, presenting many achievements and, at the same time, great challenges throughout its history. Created in 1973, it is configured as an effective public policy in the Brazilian Health System (In Portuguese, *Sistema* Único *de Saúde* - SUS) with regard to decentralization, universality and equity^(1)^.

Despite the high demand and the complexity of the vaccination schedule, Brazil stands out for offering a wide variety of vaccines free of charge, ensuring access to immunization for different age groups and specific groups. This offer is challenged by territorial diversity and the need to update processes due to epidemiological changes, requiring ongoing education to qualify professionals and adapt resources, ensuring adequate vaccines for users^(2)^.

Vaccination is an important public health strategy for preventing diseases and reducing morbidity and mortality. A major problem that has occurred in Brazil and around the world is the loss of credibility and reliability of immunobiological agents, which has generated impacts that in the medium term seriously affect the population, especially in Brazil^(3)^.

In light of the anti-vaccine movement and fake news related to immunobiological agents, Brazil is facing a public health problem that has taken on greater proportions during the COVID-19 pandemic. The dissemination of misleading information on social media and information outlets about vaccines in the Brazilian context, intensified by political polarization, increased in the first months of the pandemic, negatively impacting the population’s adherence to vaccination campaigns and social isolation measures^(4)^.

In recent years, records in the Primary Care Information System (e-SUS AB) indicate a decline in vaccination coverage among children in Brazil. Survey conducted to monitor coverage of the inactivated polio 1, 2 and 3 vaccine (IPV), between 2011 and 2021, replacing the bivalent oral polio vaccine, highlighted the drop in vaccination coverage throughout this period in all regions of the country, with a predominance in the north and northeast, especially during the pandemic, highlighting the places with greater social vulnerability, with an imminent risk of reintroduction of the wild virus and with an urgent need for interventions to strengthen the health system^(5)^.

A recent review confirmed that the population’s adherence to vaccination has decreased significantly since then, a national trend that continues to this day. The devaluation of the importance of vaccines may be linked to the population’s limited awareness of the danger of vaccine-preventable diseases that are often controlled or have mild symptoms. Moreover, the spread of incorrect information about the effectiveness of vaccines and reports of adverse events also contribute to this perception^(6)^.

During the pandemic, in addition to the lack of encouragement for vaccination and the dissemination of news without scientific evidence by the Federal Government, social isolation measures limited health units’ activities, some of which were intended for disease testing and management. This pandemic context possibly compromised other primary prevention activities, such as vaccination^(7)^.

In Rio de Janeiro, between 2006 and 2016, the average vaccination coverage of the Bacillus Calmette-Guérin (BCG), IPV, and measles, mumps, rubella (MMR) vaccines was 90.6%, 107% and 102.3%, respectively. In 2016, BCG and IPV recorded the lowest rates in the period: 81.1% and 91%. MMR had the worst coverage in 2013, with 77.1% of children up to 1 year old vaccinated. Nationally, there was a decrease in vaccination of these three vaccines over the years: BCG reduced by 0.9% per year, IPV by 1.3%, and MMR showed the greatest reduction, with 2.7% per year^(5)^. In response to this problem, the *Fundação Oswaldo Cruz* proposed the “Project for the Reconquest of High Vaccination Coverage”, which has been implemented since 2021 through the partnership between PNI and the Brazilian Society of Immunizations^(8,9)^.

Given the context presented, professionals from Family Health Strategy (FHS) in a highly vulnerable region of the city of Rio de Janeiro conducted a survey in their territory and developed practices aimed at improving vaccination indicators, achieving success. Considering the relevance of child vaccination actions for controlling morbidity and mortality, ratified in the United Nations Sustainable Development Goals in 2015^(10)^, it was decided to present initiatives aimed at increasing vaccination uptake in this group. Thus, this article aimed to report the experience of planning and implementing practices to increase vaccination coverage for children up to 2 years old in a *favela* in Rio de Janeiro.

## METHODOLOGY

This is a professional experience report study that addresses the planning and implementation of practices to increase vaccination coverage for children in a socially vulnerable area called “*Parque Proletário de Vigário Geral*”, located in the far north of the city of Rio de Janeiro. It was structured in two phases, reflecting the authors’ professional experience when describing the different stages that took place between June and November 2023. These stages include the planning and execution of strategies to expand vaccination coverage for vulnerable populations living in *favelas*. The actions were conducted by two FHS teams, composed of two nurses, two physicians, three nursing technicians and 12 community health workers (CHWs).

This report establishes correlations between the elements of care practice and the conceptual foundations that support the interventions carried out, offering a critical analysis of the strategies employed in facing the specific challenges of this scenario. To better portray the experience, two distinct stages were established.

### Stage 1 - Planning

Initial planning took into account existing structure and care model, emphasizing the importance of strategically allocating health units and organizing actions in the territory, especially those focused on prevention and health promotion. It was essential to delimit the territory and strengthen the bonds of belonging between users and healthcare services of the reference unit.

One of the fundamental tools used in this process was the Epidemiological Intelligence Center (EIC) of the City of Rio de Janeiro, which can be accessed through the public online platform called the Rio Epidemiological Observatory (EpiRio)^(11)^.

EIC emerged from the experiences accumulated by the Health Emergency Operations Center (COE-COVID-19). It was created by the Municipal Health Department (MHD) in January 2021 as part of the response to the pandemic. Officially launched in March 2022, with the support of the Pan American Health Organization and integrated with the Rio Operations Center (In Portuguese, *Centro de Operações do Rio* - COR), EIC is coordinated by the MHD Health Surveillance Superintendence. It monitors several public health indicators in the state capital, offering strategic support for formulating health policies.

Among the tools provided by EIC, GeoVacina Rio stood out, as it was essential for identifying factors related to low vaccination adherence. The system allowed for the georeferencing of vaccination coverage in different regions of the city, identifying specific locations where children with delayed vaccination status lived. This data facilitated health teams’ work, enabling nurses to systematize information in spreadsheets and carry out daily monitoring of children with delayed vaccinations. This integrated use of technology was crucial for directing actions and achieving planning objectives.

### Stage 2 - Execution

The identification of cases of delayed vaccination gave rise to actions carried out by CHWs, who worked directly in the territory actively searching for children aged 0 to 2 years to check their vaccination records. To optimize communication and records among professionals, a group was created in a messaging application, allowing CHWs to send photos of records for immediate analysis.

Among the strategies implemented, “D-Days” of vaccination stood out, with health units open on Saturdays to update vaccination records, conduct pediatric consultations and administer all vaccines on the updated schedule. Complementing these actions, Family Health teams used cell phones with messaging applications to facilitate contact with users in their areas, in addition to visiting local daycare centers, expanding the reach and effectiveness of the initiatives.

As the study did not involve primary data collection, it was not necessary to seek approval from the Research Ethics Committee.

## RESULTS

During the first implementation strategy, nurses and nursing technicians analyzed the information and identified possible gaps in the vaccination schedule. In cases where records were out of date, children’s guardians were called to the health unit to regularize the situation and to have a conversation to clarify the reasons that led them not to vaccinate the children under their responsibility. This conversation was conducted using sensitive listening, seeking to fully understand what users were expressing, both verbally and non-verbally, and asking genuine questions to deepen understanding, without giving the impression of judgment or rushing to reach an answer.

During “D-Day” vaccination events, fun and educational activities were carried out to create an attractive and welcoming environment, with games and professionals dressed as children’s characters and superheroes. This approach reduced children’s fear and encouraged them to join the campaign. In addition, as part of the health promotion initiatives, oral hygiene kits were distributed to the children present, containing a toothbrush, toothpaste and guidance on basic oral healthcare. This integration of health actions expanded the scope of the intervention, promoting not only immunization, but also other aspects of child health.

The use of digital messaging apps has played a crucial role in strengthening the bond between healthcare professionals and the community. It has made it possible to send personalized reminders about vaccination, schedule follow-ups more conveniently and organize home vaccination in specific cases, all in an agile and efficient manner. This resource contributed significantly to expanding vaccination coverage, ensuring that care was provided more quickly and in line with users’ needs, in addition to reducing access barriers and promoting more humanized care.

Finally, educational activities were expanded during visits to daycare centers in the region, including meetings with pedagogical coordinators. These meetings aimed not only to clarify doubts about early childhood vaccines, but also to raise awareness among caregivers about the importance of vaccination as an essential protective measure, considering children’s vulnerability. The coordination with daycare managers helped to mobilize caregivers to attend health units, strengthening engagement in vaccination campaigns.

During the execution of activities, it was possible to implement measures to protect, welcome and strengthen the bond between the health team and the community. Many guardians expressed appreciation for the attention they received, when they realized that their children’s health was being monitored and that the FHS team showed concern by carrying out active searches directly in the territory. These interactions became opportune moments to explain the importance of vaccination, how it works and associated benefits. The exchange of telephone contacts between guardians and the health team was an effective strategy that helped to build credibility in relation to vaccination, ensuring a channel of communication in case of adverse events related to immunization.

The actions carried out made it possible to achieve 100% coverage and update the vaccination records of all children aged 0 to 2 years registered at the unit, based on spreadsheets generated by the electronic medical record and the Geovacina Rio platform.

The health unit where actions were carried out received a certificate as “health unit with the best vaccination coverage in 2023”, for achieving the immunization indicators stipulated by the city of Rio de Janeiro. This recognition was published in regional news media, recognizing the Vigário Geral neighborhood as the most immunized in 2023, according to municipal indicators.

## DISCUSSION

The implementation of integrated strategies to expand vaccination coverage demonstrated significant results in a vulnerable territory. It included the active identification of vaccination gaps, the use of sensitive listening to engage caregivers, educational activities in daycare centers and recreational events on “D-Day” of vaccination. Digital tools facilitated scheduling and reduced access barriers, while active outreach and strengthening community ties promoted adherence. These actions ensured the vaccination of 100% of registered children aged 0 to 2 years, consolidating the impact of health promotion strategies and generating public recognition for the excellence of the unit.

The advance in vaccination coverage was reported in the local press and on television by the City Hall of Rio de Janeiro. According to the city hall, an increase in the availability of the main vaccines for children in this age group was recorded in the municipal health network. In addition, according to the Ministry of Health^(12)^, vaccination coverage grew by ten percentage points between 2022 and 2023, interrupting the downward trend observed in previous years. Among the highlights, in the city of Rio de Janeiro, IPV showed an increase of 11.83 percentage points; the human rotavirus G1P1 [8] vaccine (attenuated) grew by 11.43 percentage points; and the adsorbed diphtheria, tetanus, pertussis, hepatitis B (recombinant), and *Haemophilus influenzae* B (conjugate) vaccine grew by 11.78 percentage points.

The Vigário Geral neighborhood achieved 100% vaccination coverage for children up to 2 years of age in 2023. This result was a consequence of the intense epidemiological surveillance work carried out by nurses, nursing technicians and CHWs from the Basic Health Unit located in the neighborhood, implementing interventions that guaranteed disease prevention through user vaccination.

The community of Vigário Geral, located in northern Rio de Janeiro, has a history characterized by popular occupation and urban and social challenges typical of Rio de Janeiro communities. Its emergence dates back to the beginning of the 20^th^ century, when there was uncontrolled growth in peripheral areas and the arrival of workers attracted by the proximity of industrial and railway centers. Over the years, the area has dealt with issues such as poverty, violence and lack of basic infrastructure, but it has also established itself as an icon of resistance and organization among its residents^(13)^.

The growth of similar occupations, called “substandard agglomerations”, has become common in large urban centers, especially in southeastern Brazil, reflecting the persistence of social inequality in the country. These areas, often monitored as temporary housing solutions, are characterized by structural precariousness, highlighting challenges in urbanization and access to basic rights, despite advances in the region’s economic and social indicators^(14)^.

Residents’ health conditions are deeply influenced by socioeconomic and cultural factors, such as social class, gender, race, income, sanitation, and access to services. These social determinants directly affect vaccination uptake, with low education and income often being associated with lack of knowledge about vaccines. Vaccine hesitancy is lower among individuals with higher education, while negative experiences with reception, structural problems, religious beliefs, and safety concerns drive the population away. Strengthening reception in healthcare services is an essential strategy to encourage adherence and promote self-care^(15)^.

The act of welcoming requires more than just providing services, as it demands the practice of sensitive listening, which transcends the simple act of listening. Sensitive listening is based on empathy, recognizing the freedom and value of each person without judgment or comparison. This holistic approach seeks to understand the other in their entirety as a human being, promoting a bond of trust that can transform the relationship between healthcare professionals and the community, facilitating access to vaccines and strengthening the relationship between care and autonomy^(16)^.

In view of this, Primary Health Care (PHC) teams have integrated surveillance functions in urban, rural and remote areas, using models such as data aggregation systems, control panels and digital surveillance, which played a relevant role during COVID-19. Strategies such as active epidemiological surveillance and active search have been enhanced by the integration of information and healthcare technologies (IHCTs), such as the use of social networks, including Instagram^®^, to disseminate information about vaccination. These practices expand the reach of health actions by facilitating the production, storage, transmission and secure access to information, demonstrating the importance of technological innovation in promoting health^(17)^.

In Brazil, the SUS information policy, intensified in the 2000s, consolidated the use of IHCTs to maintain coordinated and updated information systems. In PHC, considered the gateway and coordinator of care in the Health Care Network, these technologies transformed the work process, allowing greater effectiveness in reaching users, improvements in teaching and learning, and more accurate clinical decisions. Furthermore, IHCTs have facilitated the construction of collective diagnoses on territories, strengthening healthcare services’ response capacity^(18)^.

The presence of new technologies is a reality, and has been used as a tool in the process of synthesis, dissemination and application of knowledge, such as Telehealth, the University Telemedicine Network and the *Universidade Aberta do Sistema* Único *de Saúde*. The practical application of knowledge promotes direct improvements in user health, favoring the provision of more effective products and services and strengthening the health system. Among the most used applications for mobile devices are Telegram^®^, Kakaotalk^®^ and WhatsApp^®(19)^.

WhatsApp^®^ stands out as a platform widely used worldwide for instant messaging that allows the sharing of different types of media, including texts, images, videos, documents, locations and voice calls. This feature offers quick and easy access to a variety of information, including information related to health^(19)^.

During the pandemic, the use of the application in PHC was a strategy that established new ways of managing work processes and healthcare, from a communicative, active and synchronous perspective considering the problems identified in care settings, such as the need to disseminate qualified information, in a context in which fake news or misleading information was disseminated^(20)^. They represent a relevant concern in health, especially due to the dissemination of misleading information about care, intensified during the COVID-19 pandemic.

During the pandemic, misinformation has been detrimental to home and institutional care, putting lives at risk by compromising adherence to public health measures. In response, in 2018, the Ministry of Health implemented a space on its website and social media dedicated to clarifying information based on scientific evidence, seeking to combat the negative impacts of messaging apps on the spread of rumors about diseases such as yellow fever, flu and measles. The spread of misleading information has further aggravated the decline in vaccination rates, driving users away from health units and making epidemiological control more difficult.

Even before the pandemic, ministerial studies already indicated a reduction in vaccine adherence among children, a problem that became more acute and led to vaccine hesitancy related to lack of knowledge about vaccines, fear of adverse events and side effects, and underestimation of the lethality of vaccine-preventable diseases, actions motivated by fake news^(21)^. In Malaysia, social media access was linked to vaccine hesitancy, with an increase in the number of mothers considering third-party opinions, the internet, and having doubts about the effectiveness of the vaccine^(22)^.

Brazilian studies have shown that the lack of knowledge about vaccination among parents and family members contributes to an incomplete vaccination schedule. However, professional action in disseminating information through health literacy has the potential to increase adherence and knowledge for disease prevention^(23,24)^.

Although other factors also contribute to inappropriate behavior, it is necessary to consider especially those people who do not have equal and equitable access to healthcare, as recommended by SUS. Sharing fake news is identified as one of the main reasons for non-acceptance of preventive measures and care recommended by science for the benefit of health around the world^(25)^.

Based on practices established by the health team in this territory, it was possible to make improvements in vaccination coverage, mitigating hesitancy and the reduction in vaccination coverage, serious problems since the emergence of anti-vaccine movements during the pandemic caused by the coronavirus^(26)^.

Furthermore, in Brazil, other strategies also stand out in recent literature. A study published in 2024 analyzed initiatives to increase vaccination coverage in PHC in a city in Pará. The research indicated that the success in achieving vaccination coverage was attributed to the training of teams, especially CHWs. This training aimed to provide technical information to raise awareness among families about the importance of vaccination. This approach allowed healthcare professionals to acquire skills to effectively approach families, clarifying doubts and dispelling myths about vaccines^(27)^.

Another point highlighted by the study was the active search for people who had previously been identified as absent from healthcare services. This personalized strategy proved effective in reaching individuals who did not attend vaccination campaigns, demonstrating the health team’s commitment to ensuring access to the recommended vaccines. Additionally, making vaccination available outside business hours was another important measure, considering the difficulties faced by some people in accessing services during regular business hours^(27)^.

Similarly, a study conducted in Minas Gerais reported the importance of specific strategies to increase vaccination coverage. The research described the implementation of workshops organized in four stages: motivational moment; contextual core; integrative/planning core; and integrative/results core. These workshops not only highlighted local realities and the need for improvement in immunization processes, but also promoted the development of effective strategies. As a result, there was an increase in vaccination coverage, demonstrating the effectiveness of the methodology used^(28)^.

The results from 51 experiences from all Brazilian regions on the topic of immunization, 19 from the southeast, 14 from the south, 12 from the northeast, two from the north, and four from the center-west, indicate that low vaccination coverage is a major challenge faced by healthcare throughout Brazil. The most frequent successful experiences for immunization were collective guidance, extramural vaccination, extended opening hours and training of healthcare professionals^(29)^.

The assessment of the repercussions of a ministerial intervention called “Action Plan: Vaccination Strategy at the Borders” and strategies for linking users through health education and adapting flows to promote access were identified in municipalities that were successful in improving vaccination indicators.

## CONCLUSION

The report presented showed that the practices used by FHS played a fundamental role in increasing vaccination coverage (in the territory) in the Vigário Geral *favela*, standing out as a model of excellence in meeting vaccination targets, especially in children up to 2 years of age.

Due to their vulnerability, Rio de Janeiro’s *favelas* require qualified epidemiological responsibility. Well-planned and executed interventions, combined with the strategic use of new technologies, and collaboration between healthcare professionals and the community have proven effective in reversing the decline in immunization.

Given the challenge of fake news for public health, the experience reported highlights the importance of robust health communication and education strategies to ensure adherence to preventive measures and user trust in healthcare services. Reports like this can help replicate successful strategies and, in doing so, promote health and protect against vaccine-preventable diseases.

## Data Availability

Not applicable. This study is based on an experience report and did not generate data available for sharing.
