# Supplementary material for: Health team practices to improve vaccination coverage of children in a favela
Source: Rev Esc Enferm USP. 2025 Jun 6;59:e20240337. doi: 10.1590/1980-220X-REEUSP-2024-0337en (PMC12172670; doi:10.1590/1980-220X-REEUSP-2024-0337en)
Supplement: Translation

# Translation: Práticas de equipe de saúde para melhoria da cobertura vacinal de crianças em uma favela

**DOI:** 10.1590/1980-220X-REEUSP-2024-0337pt

**Authors:** Gean Mascaranhas Gomes, Giulia Gazineo Trindade Assis, Sandra Cristina de Souza Borges Silva, Liana Amorim Corrêa Trotte, Marluci Andrade Conceição Stipp

**Affiliations:** 1 Universidade Federal do Rio de Janeiro, Centro de Ciências da Saúde, Escola de Enfermagem Anna Nery, Rio de Janeiro, RJ, Brasil.; 2 Universidade do Estado do Rio de Janeiro, Faculdade de Enfermagem, Departamento de Enfermagem Materno-Infantil, Rio de Janeiro, RJ, Brasil.

**Keywords:** Cobertura Vacinal, Saúde da Criança, Gestão em Saúde, Atenção Primária à Saúde, Enfermagem

## Abstract

**Introdução:**

Atualmente, o Programa Nacional de Imunizações enfrenta inúmeros desafios, como a hesitação vacinal, a disseminação de *fake news* e os impactos da COVID-19, especialmente em comunidades vulneráveis, onde barreiras sociais e estruturais afetam a cobertura vacinal.

**Objetivo:**

Relatar a experiência de planejamento e implementação de práticas para ampliar a cobertura vacinal de crianças de até 2 anos em uma favela do Rio de Janeiro.

**Método:**

Relato de experiência realizada entre junho e novembro de 2023, estruturada em duas etapas: planejamento, com uso do GeoVacina Rio para identificar lacunas vacinais; e execução, que incluiu busca ativa, “Dias D” de vacinação, atividades educativas e comunicação digital com os responsáveis.

**Resultados:**

Foi alcançado 100% de cobertura vacinal das crianças cadastradas, fortalecendo o vínculo com a comunidade e reduzindo barreiras de acesso por meio de estratégias integradas e humanizadas.

**Conclusão:**

O uso de tecnologia, educação em saúde e acolhimento humanizado se mostrou eficaz na ampliação da cobertura vacinal em territórios vulneráveis, servindo como modelo para outras localidades.

## INTRODUÇÃO

O Programa Nacional de Imunizações (PNI), coordenado pelo Ministério da Saúde, com ações compartilhadas com as secretarias de saúde estaduais e municipais, completou 51 anos de trajetória em 2024, apresentando muitas conquistas e, ao mesmo tempo, grandes desafios ao longo de sua história. Criado em 1973, configura-se como uma política pública eficaz no Sistema Único de Saúde (SUS) no que tange à descentralização, universalidade e equidade^(1)^.

Apesar da alta demanda e da complexidade do calendário vacinal, o Brasil destaca-se por oferecer gratuitamente uma ampla variedade de vacinas, garantindo acesso à imunização para diferentes faixas etárias e públicos específicos. Essa oferta é desafiada pela diversidade territorial e pela necessidade de atualizar processos devido a mudanças epidemiológicas, exigindo educação permanente para qualificar profissionais e adequar recursos, assegurando vacinas adequadas aos usuários^(2)^.

A vacinação é uma importante estratégia de saúde pública na prevenção de doenças e redução de morbimortalidades. Um grande problema que tem ocorrido no Brasil e no mundo é sua descredibilização e perda da confiabilidade nos imunobiológicos, o que tem gerado impactos que em médio prazo afetam gravemente a população, principalmente no Brasil^(3)^.

Tendo em vista o movimento antivacina e as *fake news* relacionadas aos imunobiológicos, o Brasil enfrenta um problema de saúde pública que assumiu maiores proporções durante o período pandêmico da COVID-19. A disseminação de informações enganosas em redes sociais e veículos de informação sobre vacinas no contexto brasileiro, intensificada pela polarização política, aumentou nos primeiros meses da pandemia, impactando negativamente a adesão da população às campanhas de vacinação e às medidas de isolamento social^(4)^.

Nos últimos anos, os registros no Sistema de Informação da Atenção Básica (e-SUS AB) sinalizam o declínio da cobertura vacinal entre crianças no Brasil. Levantamento realizado para monitorar a cobertura da vacina poliomielite 1, 2 e 3 (inativada) (VIP), entre 2011 e 2021, em substituição à vacina oral bivalente contra a poliomielite, evidenciou a queda da cobertura vacinal ao longo desse período em todas as regiões do país, com predominância do Norte e do Nordeste, especialmente durante a pandemia, destacando-se os locais com maior vulnerabilidade social, com risco iminente de reintrodução do vírus selvagem e com necessidade urgente de intervenções para fortalecer o sistema de saúde^(5)^.

Revisão recente corroborou que a adesão da população à vacinação diminuiu significativamente desde então, tendência nacional que se mantém até os dias atuais. A desvalorização da importância das vacinas pode estar ligada à consciência limitada da população sobre o perigo das doenças imunopreveníveis que frequentemente são controladas ou apresentam sintomas brandos. Além disso, a propagação de informações incorretas sobre a efetividade das vacinas e as reportagens sobre eventos adversos também contribuem para essa percepção^(6)^.

Ainda durante a pandemia, além da falta de estímulo à vacinação e da difusão de notícias sem comprovações científicas pelo Governo Federal, as medidas de isolamento social limitaram as atividades das unidades de saúde, sendo algumas destinadas à testagem e manejo da doença. Esse contexto pandêmico possivelmente comprometeu outras atividades de prevenção primária, como a vacinação^(7)^.

No Rio de Janeiro, entre 2006 e 2016, a cobertura vacinal média das vacinas Bacilo Calmette-Guérin (BCG), VIP, e sarampo, caxumba, rubéola (tríplice viral) era de 90,6%, 107% e 102,3%, respectivamente. Em 2016, a BCG e a VIP registraram as menores taxas no período: 81,1% e 91%. A tríplice viral teve a pior cobertura em 2013, com 77,1% das crianças de até 1 ano vacinadas. Nacionalmente, observou-se uma queda na vacinação dessas três vacinas ao longo dos anos: a BCG reduziu 0,9% ao ano, a VIP, 1,3%, e a tríplice viral apresentou a maior redução, com 2,7% ao ano^(5)^. Em resposta a essa problemática, a Fundação Oswaldo Cruz propôs o “Projeto pela Reconquista das Altas Coberturas Vacinais”, que vem sendo implementado desde 2021 pela parceria entre o PNI e a Sociedade Brasileira de Imunizações^(8,9)^.

Diante do contexto apresentado, profissionais da Estratégia Saúde da Família (ESF) de uma região altamente vulnerável da cidade do Rio de Janeiro fizeram um levantamento em seu território e desenvolveram práticas direcionadas à melhoria dos indicadores de vacinação, logrando êxito. Considerando a relevância das ações de vacinação das crianças para o controle da morbimortalidade, ratificadas nos Objetivos de Desenvolvimento Sustentável da Organização das Nações Unidas em 2015^(10)^, optou-se por apresentar as iniciativas voltadas à ampliação da adesão à vacinação nesse grupo. Assim, o objetivo deste artigo foi relatar a experiência de planejamento e implementação de práticas para ampliar a cobertura vacinal de crianças de até 2 anos em uma favela do Rio de Janeiro.

## METODOLOGIA

Trata-se de estudo do tipo relato de experiência profissional, que aborda o planejamento e a implementação de práticas para o aumento da cobertura vacinal de crianças em um território de vulnerabilidade social denominado “Parque Proletário de Vigário Geral”, situado no extremo norte do município do Rio de Janeiro. Foi estruturado em duas fases, refletindo a experiência profissional dos autores ao descrever as distintas etapas decorridas no período entre junho e novembro de 2023. Essas etapas compreendem o planejamento e a implementação de estratégias para a ampliação da cobertura vacinal em populações vulneráveis residentes em favela. As ações foram conduzidas por duas equipes da ESF, compostas por dois enfermeiros, dois médicos, três técnicos de enfermagem e 12 agentes comunitários de saúde (ACS).

Este relato estabelece correlações entre os elementos da prática assistencial e os fundamentos conceituais que embasam as intervenções realizadas, oferecendo uma análise crítica das estratégias empregadas no enfrentamento dos desafios específicos desse cenário. Para melhor retratar a experiência vivenciada, foram estabelecidas duas etapas distintas.

### Etapa 1-Planejamento

O planejamento inicial considerou a estrutura e o modelo assistencial existentes, enfatizando a importância da alocação estratégica das unidades de saúde e da organização das ações no território, especialmente aquelas voltadas à prevenção e promoção da saúde. Foi essencial delimitar o território e fortalecer os vínculos de pertencimento entre os usuários e os serviços de saúde da unidade de referência.

Uma das ferramentas fundamentais utilizadas nesse processo foi o Centro de Inteligência Epidemiológica (CIE) da Prefeitura do Rio de Janeiro, que pode ser acessado pela plataforma pública *online* denominada Observatório Epidemiológico do Rio (EpiRio)^(11)^.

O CIE surgiu a partir das experiências acumuladas pelo Centro de Operações de Emergência em Saúde (COE-COVID-19). Foi criado pela Secretaria Municipal de Saúde (SMS) em janeiro de 2021 como parte da resposta à pandemia. Lançado oficialmente em março de 2022, com o apoio da Organização Pan-Americana da Saúde e integrado ao Centro de Operações do Rio (COR), o CIE é coordenado pela Superintendência de Vigilância em Saúde da SMS. Monitora diversos indicadores de saúde pública na capital do estado, oferecendo suporte estratégico para a formulação de políticas de saúde.

Entre as ferramentas disponibilizadas pelo CIE, destacou-se a GeoVacina Rio, que foi fundamental para a identificação de fatores relacionados à baixa adesão vacinal. O sistema permitiu o georreferenciamento da cobertura vacinal nas diferentes regiões da cidade, identificando localidades específicas onde residiam crianças com situação vacinal em atraso. Esses dados facilitaram o trabalho das equipes de saúde, possibilitando que os enfermeiros sistematizassem informações em planilhas e realizassem o acompanhamento diário das crianças com vacinas atrasadas. Esse uso integrado da tecnologia foi determinante para direcionar as ações e alcançar os objetivos do planejamento.

### Etapa 2-Execução

A identificação dos casos de atraso vacinal deu início às ações conduzidas pelos ACS, que atuaram diretamente no território com busca ativa de crianças de 0 a 2 anos para a verificação das cadernetas de vacinação. Para otimizar a comunicação e os registros entre os profissionais, foi criado um grupo em um aplicativo de mensagens, permitindo o envio de fotos das cadernetas pelos ACS para análise imediata.

Entre as estratégias implementadas, destacaram-se os “Dias D” de vacinação, com unidades de saúde abertas aos sábados para atualização das cadernetas vacinais, realização de consultas pediátricas e aplicação de todas as vacinas do calendário atualizado. Complementando essas ações, as equipes de Saúde da Família utilizaram aparelhos celulares com aplicativos de mensagens para facilitar o contato com os usuários de suas áreas, além de realizarem visitas às creches locais, ampliando o alcance e a efetividade das iniciativas.

Como o estudo não envolveu a coleta de dados primários, não foi necessária a apreciação do Comitê de Ética em Pesquisa.

## RESULTADOS

Durante a primeira estratégia da execução, enfermeiros e técnicos de enfermagem analisavam as informações e identificavam possíveis lacunas no esquema vacinal. Nos casos de cadernetas desatualizadas, os responsáveis pelas crianças eram convocados à unidade de saúde para regularização e uma conversa para esclarecimento dos motivos que os levaram a não vacinar as crianças sob sua responsabilidade. Essa conversa era realizada utilizando uma escuta sensível, buscando compreender plenamente o que o usuário estava expressando, tanto em termos verbais quanto não verbais, e fazendo perguntas genuínas para o aprofundamento da compreensão, sem dar a impressão de julgamento ou pressa para chegar à resposta.

Já durante os eventos do “Dia D” de vacinação, atividades lúdicas e educativas foram realizadas para criar um ambiente atraente e acolhedor, com brincadeiras e profissionais fantasiados de personagens infantis e super-heróis. Essa abordagem reduziu o medo das crianças e incentivou a adesão à campanha. Além disso, como parte das iniciativas de promoção da saúde, foram distribuídos *kits* de higiene bucal para as crianças presentes, contendo escova, pasta de dentes e orientações sobre cuidados básicos com a saúde bucal. Essa integração de ações de saúde ampliou o alcance da intervenção, promovendo não apenas a imunização, mas também outros aspectos da saúde infantil.

O uso da ferramenta digital de aplicativos de mensagens desempenhou um papel crucial no fortalecimento do vínculo entre os profissionais de saúde e a comunidade. Tornou possível enviar lembretes personalizados sobre a vacinação, agendar retornos com maior praticidade e organizar a vacinação no domicílio em casos específicos, tudo de forma ágil e eficiente. Esse recurso contribuiu significativamente para ampliar a cobertura vacinal, garantindo que os atendimentos ocorressem de maneira mais rápida e alinhada às necessidades dos usuários, além de reduzir as barreiras de acesso e promover o atendimento mais humanizado.

Por fim, nas visitas às creches do território, as ações educativas foram ampliadas, envolvendo reuniões com os coordenadores pedagógicos. Esses encontros visaram não apenas esclarecer dúvidas sobre as vacinas da primeira infância, mas também fomentar a conscientização entre os responsáveis sobre a relevância da vacinação como medida essencial de proteção, considerando a vulnerabilidade das crianças. A articulação com os gestores das creches contribuiu para mobilizar os responsáveis a comparecerem às unidades de saúde, fortalecendo o engajamento nas campanhas de vacinação.

Durante a execução das atividades, foi possível implementar medidas de proteção, acolhimento e fortalecimento do vínculo entre a equipe de saúde e a comunidade. Muitos responsáveis demonstraram reconhecimento pela atenção recebida, ao perceberem que a saúde de seus filhos estava sendo monitorada e que a equipe da ESF se mostrava preocupada ao realizar a busca ativa diretamente no território. Essas interações tornaram-se momentos oportunos para explicar a importância da vacinação, seu funcionamento e os benefícios associados. A troca de contatos telefônicos entre os responsáveis e a equipe de saúde foi uma estratégia eficaz que ajudou a construir credibilidade em relação à vacinação, assegurando um canal de comunicação caso surgissem eventos adversos relacionados à imunização.

As ações realizadas permitiram alcançar 100% de cobertura e atualizar a caderneta vacinal de todas as crianças com idades de 0 a 2 anos cadastradas na unidade, a partir das planilhas geradas pelo prontuário eletrônico e da plataforma Geovacina Rio.

A unidade de saúde onde foram realizadas as ações recebeu um certificado de “unidade de saúde com melhor cobertura vacinal no ano de 2023”, pelo alcance dos indicadores de imunização estipulados pelo município do Rio de Janeiro. Esse reconhecimento foi publicado nas mídias informativas regionais, reconhecendo o bairro Vigário Geral como o mais imunizado de 2023, de acordo com os indicadores municipais.

## DISCUSSÃO

A implementação de estratégias integradas para ampliar a cobertura vacinal demonstrou resultados significativos em um território vulnerável. Incluiu a identificação ativa de lacunas vacinais, uso de escuta sensível para engajamento dos responsáveis, ações educativas em creches e eventos lúdicos no “Dia D” de vacinação. Ferramentas digitais facilitaram o agendamento e reduziram barreiras de acesso, enquanto a busca ativa e o fortalecimento do vínculo com a comunidade promoveram a adesão. Essas ações garantiram a vacinação de 100% das crianças de 0 a 2 anos cadastradas, consolidando o impacto das estratégias na promoção da saúde e gerando reconhecimento público pela excelência da unidade.

O avanço da cobertura vacinal foi veiculado na imprensa local e em mídias televisivas pela Prefeitura do Rio de Janeiro. De acordo com a prefeitura, foi registrado na rede municipal de saúde um aumento na disponibilidade dos principais imunizantes para crianças dessa faixa etária. Além disso, de acordo com o Ministério da Saúde^(12)^, as coberturas vacinais tiveram um crescimento de dez pontos percentuais entre 2022 e 2023, interrompendo a tendência de queda observada nos anos anteriores. Entre os destaques, no município do Rio de Janeiro, a VIP apresentou um aumento de 11,83 pontos percentuais; a vacina rotavírus humano G1P1 [8] (atenuada) cresceu 11,43 pontos percentuais; e a vacina adsorvida difteria, tétano, pertussis, hepatite B (recombinante) e *Haemophilus influenzae* B (conjugada) cresceu 11,78 pontos percentuais.

O bairro Vigário Geral alcançou 100% de cobertura vacinal de crianças de até 2 anos de idade em 2023. Esse resultado foi consequência do intenso trabalho de vigilância epidemiológica realizado pelos enfermeiros, técnicos de enfermagem e ACS da Unidade Básica de Saúde localizada no bairro, implementando intervenções que garantiram a prevenção de doenças por meio da vacinação dos usuários.

A comunidade de Vigário Geral, situada na zona Norte do Rio de Janeiro, possui uma trajetória caracterizada pela ocupação popular e por desafios urbanos e sociais típicos das comunidades cariocas. Seu surgimento remonta ao início do século XX, quando houve um crescimento descontrolado das áreas periféricas e a chegada de trabalhadores atraídos pela proximidade de centros industriais e ferroviários. Com o passar dos anos, a área lidou com questões como pobreza, violência e carência de infraestrutura fundamental, mas também se firmou como um ícone de resistência e organização entre seus moradores^(13)^.

O crescimento de ocupações semelhantes, denominadas “aglomerados subnormais”, tornou-se comum em grandes centros urbanos, especialmente no sudeste do Brasil, refletindo a persistência da desigualdade social no país. Essas áreas, muitas vezes monitoradas como soluções habitacionais temporárias, caracterizam-se pela precariedade estrutural, evidenciando desafios de urbanização e acesso a direitos básicos, apesar dos avanços nos indicadores econômicos e sociais da região^(14)^.

As condições de saúde dos moradores são profundamente influenciadas por fatores socioeconômicos e culturais, como classe social, gênero, cor, renda, saneamento e acesso a serviços. Esses determinantes sociais afetam diretamente a adesão à vacinação, sendo que a baixa escolaridade e a renda frequentemente estão associadas ao desconhecimento sobre vacinas. A hesitação vacinal é menor entre indivíduos com maior escolaridade, enquanto experiências negativas no acolhimento, problemas estruturais, crenças religiosas e preocupações com a segurança afastam a população. Fortalecer o acolhimento nos serviços de saúde é uma estratégia essencial para incentivar a adesão e promover o autocuidado^(15)^.

O ato de acolher requer mais do que disponibilizar serviços, pois demanda a prática da escuta sensível, que transcende o simples ato de ouvir. A escuta sensível baseia-se na empatia, reconhecendo a liberdade e o valor de cada pessoa sem julgamentos ou comparações. Essa abordagem holística busca compreender o outro em sua totalidade como ser humano, promovendo um vínculo de confiança que pode transformar a relação entre profissionais de saúde e a comunidade, facilitando o acesso às vacinas e fortalecendo a relação entre cuidado e autonomia^(16)^.

Diante disso, equipes da Atenção Primária à Saúde (APS) têm integrado funções de vigilância em zonas urbanas, rurais e remotas, utilizando modelos como sistemas de agregação de dados, painéis de controle e vigilância digital, que desempenharam papel relevante durante a COVID-19. Estratégias como vigilância epidemiológica ativa e busca ativa têm sido potencializadas pela integração de tecnologias de informação e cuidado em saúde (TICS), como o uso de redes sociais, incluindo o *Instagram* ^®^, para difundir informações sobre a vacinação. Essas práticas ampliam o alcance das ações de saúde ao facilitar a produção, o armazenamento, a transmissão e o acesso seguro às informações, demonstrando a importância da inovação tecnológica na promoção da saúde^(17)^.

No Brasil, a política de informação do SUS, intensificada nos anos 2000, consolidou o uso das TICS para manter sistemas de informação articulados e atualizados. Na APS, considerada a porta de entrada e coordenadora do cuidado na Rede de Atenção à Saúde, essas tecnologias transformaram o processo de trabalho, permitindo maior efetividade no alcance dos usuários, melhorias no ensino-aprendizagem e decisões clínicas mais precisas. Além disso, as TICS têm facilitado a construção de diagnósticos coletivos sobre os territórios, fortalecendo a capacidade de resposta dos serviços de saúde^(18)^.

A presença de novas tecnologias é uma realidade, e tem sido utilizada como ferramenta no processo de síntese, disseminação e aplicação do conhecimento, tais como o Telessaúde, a Rede Universitária de Telemedicina e a Universidade Aberta do Sistema Único de Saúde. A aplicação prática do conhecimento promove melhorias diretas na saúde dos usuários, favorecendo a disponibilização de produtos e serviços mais efetivos e fortalecendo o sistema de saúde. Entre os aplicativos mais utilizados para dispositivos móveis, estão o *Telegram* ^®^ *,* o *Kakaotalk* ^®^ e o *WhatsApp* ^®(19)^.

Destaca-se o *WhatsApp* ^®^ *,* uma plataforma amplamente utilizada no mundo para troca instantânea de mensagens que possibilita o compartilhamento de diferentes tipos de mídia, incluindo textos, imagens, vídeos, documentos, localização e chamadas de voz. Esse recurso oferece acesso ágil e facilitado a diversas informações, inclusive relacionadas à saúde^(19)^.

Durante a pandemia, a utilização do aplicativo na APS foi uma estratégia que instituiu novas formas de gestão dos processos de trabalho e dos cuidados em saúde, em uma perspectiva comunicativa, ativa e síncrona diante dos problemas identificados nos cenários assistenciais, tais como a necessidade de disseminação de informações qualificadas, em um contexto em que foram veiculadas as *fake news* ou informações inverídicas^(20)^. Representam uma preocupação relevante na saúde, especialmente pela disseminação de informações falsas sobre cuidados, intensificada durante a pandemia de COVID-19.

Durante a pandemia, a desinformação trouxe prejuízos aos cuidados domiciliários e institucionais, colocando vidas em risco ao comprometer a adesão às medidas de saúde pública. Em resposta, em 2018, o Ministério da Saúde implementou um espaço em seu *site* e redes sociais dedicado a esclarecer informações com base em evidências científicas, buscando combater os impactos negativos de aplicativos de mensagens na disseminação de boatos sobre doenças como febre amarela, gripe e sarampo. A propagação de informações falsas agravou ainda mais o declínio dos indicadores vacinais, afastando os usuários das unidades de saúde e dificultando o controle epidemiológico.

Antes mesmo do período pandêmico, os estudos ministeriais já indicavam a redução da adesão vacinal entre crianças, problemática que se agudizou e levou à hesitação vacinal relacionada ao desconhecimento sobre as vacinas, ao medo de eventos adversos e efeitos colaterais e à subestimação da letalidade das doenças imunopreveníveis, ações motivadas por *fake news* ^(21)^. Na Malásia, o acesso à rede sociais foi relacionado à hesitação vacinal, com o aumento do número de mães que consideravam opiniões de terceiros, da internet e que apresentavam dúvidas quanto à efetividade da vacina^(22)^.

Estudos brasileiros demonstraram que a fragilidade do conhecimento sobre vacinação por parte dos pais e familiares concorre para o esquema vacinal incompleto. No entanto, a atuação profissional na divulgação de informações por meio da alfabetização em saúde tem potencial para aumentar a adesão e os conhecimentos para a prevenção de doenças^(23,24)^.

Apesar de outros fatores também contribuírem para condutas inadequadas, é necessário considerar especialmente as pessoas que não têm acesso equânime e igualitário à saúde, conforme preconizado pelo SUS. O compartilhamento de *fake news* é identificado como uma das principais razões para a não aceitação de medidas preventivas e cuidados preconizados pela ciência em prol da saúde ao redor do mundo^(25)^.

A partir de práticas estabelecidas pela equipe de saúde desse território, foi possível realizar melhorias na cobertura vacinal, atenuando a hesitação e a redução da cobertura vacinal, problemas graves desde que houve o surgimento dos movimentos antivacina divulgados durante a pandemia provocada pelo coronavírus^(26)^.

Além disso, no Brasil, outras estratégias também se destacam na literatura recente. Estudo publicado em 2024 analisou iniciativas para aumentar a cobertura vacinal na APS em uma cidade do Pará. A pesquisa apontou que o sucesso no alcance da cobertura vacinal foi atribuído à capacitação das equipes, especialmente dos ACS. Essa capacitação teve como objetivo fornecer informações técnicas para sensibilizar as famílias sobre a importância da vacinação. Tal abordagem permitiu que os profissionais de saúde adquirissem habilidades para abordar as famílias de forma eficaz, esclarecendo dúvidas e dissipando mitos sobre vacinas^(27)^.

Outro ponto destacado pelo estudo foi a busca ativa de pessoas que haviam sido previamente identificadas como faltosas nos serviços de saúde. Essa estratégia personalizada mostrou-se eficaz para alcançar indivíduos que não compareceram às campanhas de vacinação, demonstrando o compromisso da equipe de saúde em garantir o acesso às vacinas recomendadas. Adicionalmente, a disponibilização da vacinação fora do horário comercial foi outra medida importante, considerando as dificuldades enfrentadas por algumas pessoas para acessar os serviços durante o expediente regular^(27)^.

De forma semelhante, estudo realizado em Minas Gerais relatou a importância de estratégias específicas para aumentar a cobertura vacinal. A pesquisa descreveu a realização de oficinas organizadas em quatro etapas: momento motivacional; núcleo contextual; núcleo integrador/planejamento; e núcleo integrador/resultados. Essas oficinas não apenas evidenciaram as realidades locais e as necessidades de melhoria nos processos de imunização, mas também promoveram o desenvolvimento de estratégias eficazes. Como resultado, houve um aumento nas coberturas vacinais, evidenciando a eficácia da metodologia empregada^(28)^.

Os resultados provenientes de 51 experiências de todas as regiões brasileiras sobre a temática de imunização, sendo 19 do Sudeste, 14, do Sul, 12, do Nordeste, dois, do Norte, e quatro, do Centro-Oeste, apontam que a baixa cobertura vacinal é um vultuoso desafio enfrentado pela saúde em todo o território brasileiro. Foram identificadas com maior frequência, nas experiências exitosas para imunização, as orientações coletivas, a vacinação extramuros, a ampliação do horário de funcionamento e a capacitação dos profissionais de saúde^(29)^.

A avaliação das repercussões da intervenção ministerial denominada “Plano de Ação: Estratégia de Vacinação nas Fronteiras” e as estratégias de vinculação dos usuários por meio da educação em saúde e de adequação dos fluxos para promover o acesso foram identificadas em municípios exitosos nas melhoras dos indicadores vacinais.

## CONCLUSÃO

O relato apresentado evidenciou que as práticas utilizadas pela ESF desempenharam papel fundamental no aumento da cobertura vacinal (no território) na favela de Vigário Geral, destacando-se como modelo de excelência no cumprimento das metas de vacinação, especialmente em crianças de até 2 anos de idade.

As favelas do Rio de Janeiro, por sua vulnerabilidade, demandam uma responsabilidade epidemiológica qualificada. Intervenções bem planejadas e executadas, combinadas ao uso estratégico de novas tecnologias, e a colaboração entre profissionais de saúde e a comunidade demonstram ser eficazes para reverter a queda na imunização.

Diante do desafio das *fake news* para a saúde pública, a experiência relatada destaca a importância de estratégias robustas de comunicação e educação em saúde, para garantir a adesão às medidas preventivas e a confiança dos usuários nos serviços de saúde. Relatos como este podem auxiliar na replicação de estratégias de sucesso e, com isso, promover a saúde e proteger contra doenças preveníveis por vacinação.

## Data Availability

Não se aplicam. Este estudo é baseado em um relato de experiência e não gerou dados disponíveis para compartilhamento.

# Translation: Prácticas del equipo de salud para mejorar la cobertura de vacunación entre los niños de una favela

**DOI:** 10.1590/1980-220X-REEUSP-2024-0337es

**Authors:** Gean Mascaranhas Gomes, Giulia Gazineo Trindade Assis, Sandra Cristina de Souza Borges Silva, Liana Amorim Corrêa Trotte, Marluci Andrade Conceição Stipp

**Affiliations:** 1 Universidade Federal do Rio de Janeiro, Centro de Ciências da Saúde, Escola de Enfermagem Anna Nery, Rio de Janeiro, RJ, Brasil.; 2 Universidade do Estado do Rio de Janeiro, Faculdade de Enfermagem, Departamento de Enfermagem Materno-Infantil, Rio de Janeiro, RJ, Brasil.

**Keywords:** Cobertura de Vacunación, Salud Infantil, Gestión en Salud, Atención Primaria de Salud, Enfermería

## Abstract

**Introducción:**

Actualmente, el Programa Nacional de Inmunizaciones enfrenta numerosos desafíos, como la reticencia a las vacunas, la difusión de noticias falsas y los impactos de la COVID-19, especialmente en comuniddes vulnerables, donde las barreras sociales y estructurales afectan la cobertura de vacunación.

**Objetivo:**

Relatar la experiencia de planificación e implementación de prácticas para ampliar la cobertura de vacunación de niños de hasta 2 años de edad en una favela de Río de Janeiro.

**Método:**

Relato de una experiencia realizada entre junio y noviembre de 2023, estructurada en dos etapas: planificación, utilizando GeoVacina Rio para identificar brechas de vacunación; y ejecución, que incluyó búsqueda activa, “Días D” de vacunación, actividades educativas y comunicación digital con los responsables.

**Resultados:**

Se logró una cobertura de vacunación del 100% en los niños registrados, fortaleciendo los vínculos con la comunidad y reduciendo las barreras de acceso mediante estrategias integrales y humanizadas.

**Conclusión:**

El uso de tecnología, educación en salud y atención humanizada se mostró eficaz para ampliar la cobertura de vacunación en territorios vulnerables, sirviendo como modelo para otras localidades.

## INTRODUCCIÓN

El Programa Nacional de Inmunización (PNI), coordinado por el Ministerio de Salud, con acciones compartidas con las secretarías de salud de los estados y municipios, cumplió 51 años en 2024, presentando muchas conquistas y, al mismo tiempo, grandes desafíos a lo largo de su historia. Creado en 1973, es una política pública eficaz dentro del Sistema Único de Salud (SUS) en términos de descentralización, universalidad y equidad^(1)^.

A pesar de la alta demanda y de la complejidad del esquema de vacunación, Brasil se destaca por ofrecer una amplia variedad de vacunas de forma gratuita, garantizando el acceso a la inmunización para diferentes grupos de edad y públicos específicos. Esta oferta se ve desafiada por la diversidad territorial y la necesidad de actualizar los procesos debido a los cambios epidemiológicos, lo que requiere una formación continua para cualificar a los profesionales y adaptar los recursos, garantizando vacunas adecuadas para los usuarios^(2)^.

La vacunación es una importante estrategia de salud pública para prevenir enfermedades y reducir la morbimortalidad. Un gran problema que ha ocurrido en Brasil y en el mundo es su descrédito y la pérdida de confiabilidad en los inmunobiológicos, lo que ha generado impactos que a mediano plazo afectan seriamente a la población, especialmente en Brasil^(3)^.

Ante el movimiento antivacunas y las *fake news* relacionadas con los inmunobiológicos, Brasil se enfrenta a un problema de salud pública que ha adquirido mayores proporciones durante el período de pandemia de COVID-19. La difusión de información engañosa en las redes sociales y en los vehículos de información sobre vacunas en el contexto brasileño, intensificada por la polarización política, aumentó en los primeros meses de la pandemia, impactando negativamente en la adhesión de la población a las campañas de vacunación y a las medidas de aislamiento social^(4)^.

En los últimos años, los registros en el Sistema de Información de Atención Primaria (e-SUS AB) han señalado una disminución de la cobertura de vacunación entre los niños en Brasil. Una encuesta realizada para monitorear la cobertura de la vacuna antipoliomielítica 1, 2 y 3 (inactivada) (IPV) entre 2011 y 2021, en sustitución de la vacuna antipoliomielítica oral bivalente, mostró una caída en la cobertura de vacunación en este período en todas las regiones del país, con predominio en el Norte y Nordeste, especialmente durante la pandemia, destacando lugares con mayor vulnerabilidad social, con riesgo inminente de reintroducción del virus salvaje y con necesidad urgente de intervenciones para fortalecer el sistema de salud^(5)^.

Una revisión reciente corroboró que la adherencia de la población a la vacunación ha disminuido significativamente desde entonces, una tendencia nacional que continúa hasta el día de hoy. La devaluación de la importancia de las vacunas puede estar relacionada con la escasa concienciación de la población sobre el peligro de las enfermedades prevenibles mediante vacunación, que a menudo se controlan o presentan síntomas leves. Además, la difusión de información incorrecta sobre la eficacia de las vacunas y los informes sobre acontecimientos adversos también contribuyen a esta percepción^(6)^.

También durante la pandemia, además de la falta de incentivo a la vacunación y de la divulgación de noticias sin evidencia científica por parte del Gobierno Federal, las medidas de aislamiento social limitaron las actividades de las unidades de salud, algunas de las cuales tenían como objetivo la realización de pruebas y el control de la enfermedad. Este contexto pandémico posiblemente puso en riesgo otras actividades de prevención primaria, como la vacunación^(7)^.

En Río de Janeiro, entre 2006 y 2016, la cobertura media de vacunación de las vacunas Bacillus Calmette-Guérin (BCG), IPV y sarampión, parotiditis y rubéola (triple vírica) fue de 90,6%, 107% y 102,3%, respectivamente. En 2016, la BCG y la IPV registraron las tasas más bajas del periodo: 81,1% y 91%. La triple vírica tuvo la peor cobertura en 2013, con un 77,1% de niños de hasta un año vacunados. A nivel nacional, se ha producido un descenso en la vacunación de estas tres vacunas a lo largo de los años: la BCG descendió un 0,9% anual, la VPI un 1,3% y la triple vírica presentó la mayor reducción, con un 2,7% anual^(5)^ En respuesta a este problema, la Fundación Oswaldo Cruz propuso el «Proyecto de Reconquista de Altas Coberturas de Vacunación», implementado desde 2021 a través de una asociación entre el PNI y la Sociedad Brasileña de Inmunizaciones^(8,9)^.

Ante este contexto, profesionales de la Estrategia de Salud de la Familia (ESF) de una región altamente vulnerable de la ciudad de Río de Janeiro realizaron un relevamiento de su territorio y desarrollaron prácticas tendientes a mejorar los indicadores de vacunación, logrando éxito. Considerando la importancia de las acciones de vacunación infantil para el control de la morbimortalidad, ratificada en los Objetivos de Desarrollo Sostenible de las Naciones Unidas en 2015^(10)^, decidimos presentar iniciativas dirigidas a aumentar la adherencia a la vacunación en este grupo. Por lo tanto, el objetivo de este artículo fue informar sobre la experiencia de planificación e implementación de prácticas para aumentar la cobertura de vacunación entre los niños de hasta 2 años en una favela de Río de Janeiro.

## METODOLOGÍA

Se trata de un informe de experiencia profesional sobre la planificación e implementación de prácticas para aumentar la cobertura de vacunación de niños en una zona socialmente vulnerable denominada «Parque Proletario de Vigário Geral», situada en el extremo norte del municipio de Río de Janeiro. Se estructuró en dos fases, reflejando la experiencia profesional de los autores en la descripción de las diferentes etapas que tuvieron lugar entre junio y noviembre de 2023. Estas etapas incluyen la planificación y ejecución de estrategias para aumentar la cobertura de vacunación entre las poblaciones vulnerables que viven en favelas. Las acciones fueron llevadas a cabo por dos equipos del FSE, formados por dos enfermeros, dos médicos, tres técnicos de enfermería y 12 agentes comunitarios de salud (ACS).

Este informe establece correlaciones entre los elementos de la práctica asistencial y los fundamentos conceptuales que sustentan las intervenciones realizadas, ofreciendo un análisis crítico de las estrategias empleadas para hacer frente a los desafíos específicos de este escenario. Para retratar mejor la experiencia, se establecieron dos etapas diferenciadas.

### Etapa 1-Planificación

La planificación inicial consideró la estructura y el modelo de atención existentes, destacando la importancia de la asignación estratégica de las unidades sanitarias y la organización de las acciones en el territorio, especialmente las dirigidas a la prevención y la promoción de la salud. Era esencial delimitar el territorio y reforzar los vínculos de pertenencia entre los usuarios y los servicios sanitarios de la unidad de referencia.

Una de las herramientas fundamentales utilizadas en este proceso fue el Centro de Inteligencia Epidemiológica (CIE) del Ayuntamiento de Río de Janeiro, al que se puede acceder a través de la plataforma pública *en* línea denominada Observatorio Epidemiológico de Río (EpiRio)^(11)^.

El CIE surgió de las experiencias acumuladas por el Centro de Operaciones de Emergencia Sanitaria (COE-COVID-19). Fue creado por la Secretaría Municipal de Salud (SMS) en enero de 2021 como parte de la respuesta a la pandemia. Lanzado oficialmente en marzo de 2022, con el apoyo de la Organización Panamericana de la Salud e integrado al Centro de Operaciones de Río (COR), el CIE es coordinado por la Superintendencia de Vigilancia en Salud de la SMS. Monitorea diversos indicadores de salud pública en la capital del estado, ofreciendo apoyo estratégico para la formulación de políticas de salud.

Entre las herramientas puestas a disposición por el CIE, destacó GeoVacina Rio, fundamental para identificar los factores relacionados con la baja aceptación de las vacunas. El sistema permitió georreferenciar la cobertura de vacunación en las diferentes regiones de la ciudad, identificando los lugares específicos donde vivían los niños con retraso en la vacunación. Estos datos facilitaron el trabajo de los equipos sanitarios, permitiendo a las enfermeras sistematizar la información en hojas de cálculo y realizar un seguimiento diario de los niños con vacunas atrasadas. Este uso integrado de la tecnología fue decisivo para dirigir las acciones y alcanzar los objetivos de la planificación.

### Etapa 2-Ejecución

La identificación de los casos de retraso en la vacunación inició las acciones llevadas a cabo por los ACS, que trabajaron directamente en la zona buscando activamente a los niños de 0 a 2 años para comprobar sus carnés de vacunación. Para optimizar la comunicación y los registros entre los profesionales, se creó un grupo en una app de mensajería, que permitía a los ACS enviar fotos de las cartillas para su análisis inmediato.

Entre las estrategias puestas en marcha destacan los «Días D» de vacunación, con unidades sanitarias abiertas los sábados para actualizar los carnés de vacunación, realizar consultas pediátricas y administrar todas las vacunas del calendario actualizado. Como complemento a estas acciones, los equipos de Salud de la Familia utilizaron teléfonos móviles con aplicaciones de mensajería para facilitar el contacto con los usuarios de sus zonas, además de realizar visitas a las guarderías locales, ampliando el alcance y la eficacia de las iniciativas.

Como el estudio no implicaba la recolección de datos primarios, no requirió la aprobación del Comité de Ética de la Investigación.

## RESULTADOS

Durante la primera estrategia de aplicación, las enfermeras y los técnicos de enfermería analizaron la información e identificaron posibles lagunas en el calendario de vacunación. En el caso de cartillas desactualizadas, los responsables por los niños fueron citados a la unidad de salud para regularización y conversación para aclarar las razones por las cuales no habían vacunado a los niños a su cargo. Esta conversación se llevó a cabo utilizando la escucha sensible, tratando de comprender plenamente lo que el usuario estaba expresando, tanto verbal como no verbalmente, y haciendo preguntas genuinas para profundizar en la comprensión, sin dar la impresión de juzgar o precipitarse en una respuesta.

Durante los actos de vacunación del «Día D» se llevaron a cabo actividades lúdicas y educativas para crear un entorno atractivo y acogedor, con juegos y profesionales disfrazados de personajes infantiles y superhéroes. Este enfoque redujo el miedo de los niños y los animó a unirse a la campaña. Además, como parte de las iniciativas de promoción de la salud, se distribuyeron *kits de* higiene bucodental a los niños presentes, que contenían cepillos de dientes, pasta dentífrica y orientación sobre cuidados básicos de salud bucodental. Esta integración de acciones sanitarias amplió el alcance de la intervención, promoviendo no solo la inmunización sino también otros aspectos de la salud infantil.

El uso de aplicaciones de mensajería digital desempeñó un papel crucial en el fortalecimiento del vínculo entre los profesionales sanitarios y la comunidad. Ha permitido enviar recordatorios de vacunación personalizados, programar citas de forma más cómoda y organizar la vacunación a domicilio en casos concretos, todo ello de forma ágil y eficiente. Este recurso contribuyó significativamente a ampliar la cobertura de vacunación, garantizando que las citas se concertaran con mayor rapidez y en función de las necesidades de los usuarios, además de reducir las barreras de acceso y promover una atención más humanizada.

Por último, se ampliaron las actividades educativas durante las visitas a las guarderías del territorio, con reuniones con los coordinadores pedagógicos. Estas reuniones tenían como objetivo no sólo aclarar dudas sobre la vacunación en la primera infancia, sino también concienciar a los cuidadores sobre la importancia de la vacunación como medida de protección esencial, teniendo en cuenta la vulnerabilidad de los niños. El contacto con los directores de guarderías ayudó a movilizar a los tutores para que acudieran a las unidades sanitarias, reforzando su compromiso con las campañas de vacunación.

En el transcurso de las actividades, fue posible aplicar medidas de protección, acogida y refuerzo del vínculo entre el equipo sanitario y la comunidad. Muchos cuidadores mostraron su agradecimiento por la atención recibida al darse cuenta de que la salud de sus hijos era objeto de seguimiento y de que el equipo del ESF mostraba preocupación realizando búsquedas activas directamente en la zona. Estas interacciones se convirtieron en momentos oportunos para explicar la importancia de la vacunación, su funcionamiento y los beneficios asociados. El intercambio de contactos telefónicos entre los responsables y el equipo sanitario fue una estrategia eficaz que ayudó a crear credibilidad en relación con la vacunación, garantizando un canal de comunicación en caso de eventos adversos relacionados con la inmunización.

Las acciones realizadas permitieron alcanzar el 100% de cobertura y actualización de la libreta de vacunación de todos los niños de 0 a 2 años registrados en la unidad, a partir de las planillas generadas por la historia clínica electrónica y la plataforma Geovacina Rio.

La unidad de salud donde se llevaron a cabo las acciones recibió un certificado como «unidad de salud con la mejor cobertura de vacunación en 2023», por alcanzar los indicadores de inmunización estipulados por el municipio de Río de Janeiro. Este reconocimiento fue publicado en los medios de comunicación regionales, reconociendo al barrio de Vigário Geral como el más inmunizado en 2023, según los indicadores municipales.

## DISCUSIÓN

La implementación de estrategias integradas para aumentar la cobertura de vacunación demostró resultados significativos en un territorio vulnerable. Incluyó la identificación activa de lagunas de vacunación, el uso de la escucha sensible para involucrar a los cuidadores, acciones educativas en guarderías y eventos divertidos sobre la vacunación «Día D». Las herramientas digitales facilitaron la programación y redujeron las barreras de acceso, mientras que la búsqueda activa y el fortalecimiento del vínculo con la comunidad promovieron la adherencia. Estas acciones lograron que el 100% de los niños inscriptos de 0 a 2 años fueran vacunados, consolidando el impacto de las estrategias de promoción de la salud y generando un reconocimiento público por la excelencia de la unidad.

El avance en la cobertura de vacunación fue divulgado en la prensa local y en la televisión por la Municipalidad de Río de Janeiro. Según la alcaldía, la red municipal de salud ha visto aumentar la disponibilidad de los principales inmunizantes para los niños de este grupo de edad. Además, según el Ministerio de Salud^(12)^, la cobertura de vacunación aumentó diez puntos porcentuales entre 2022 y 2023, interrumpiendo la tendencia a la baja observada en años anteriores. Entre los destaques, en el municipio de Río de Janeiro, la IPV presentó un aumento de 11,83 puntos porcentuales; la vacuna contra el rotavirus humano G1P1 [8] (atenuada) creció 11,43 puntos porcentuales; y la vacuna adsorbida difteria, tétano, tos ferina, hepatitis B (recombinante) y *Haemophilus influenzae* B (conjugada) creció 11,78 puntos porcentuales.

El barrio de Vigário Geral alcanzó una cobertura de vacunación del 100% de los niños de hasta 2 años en 2023. Este resultado fue consecuencia del intenso trabajo de vigilancia epidemiológica realizado por las enfermeras, técnicos de enfermería y ACS de la Unidad Básica de Salud ubicada en el barrio, implementando intervenciones que garantizaron la prevención de enfermedades mediante la vacunación de los usuarios.

La comunidad de Vigário Geral, situada en el norte de Río de Janeiro, tiene una historia caracterizada por la ocupación popular y los desafíos urbanos y sociales típicos de las comunidades cariocas. Su surgimiento se remonta a principios del siglo XX, cuando se produjo el crecimiento descontrolado de las zonas periféricas y la llegada de trabajadores atraídos por la proximidad de los centros industriales y ferroviarios. A lo largo de los años, la zona se ha enfrentado a problemas como la pobreza, la violencia y la falta de infraestructuras básicas, pero también se ha consolidado como un icono de resistencia y organización entre sus residentes^(13)^.

El crecimiento de ocupaciones similares, denominadas «aglomeraciones subnormales», se ha hecho común en los grandes centros urbanos, especialmente en el sudeste de Brasil, lo que refleja la persistencia de la desigualdad social en el país. Estas áreas, a menudo controladas como soluciones habitacionales temporales, se caracterizan por la precariedad estructural, lo que pone de relieve los desafíos de la urbanización y el acceso a los derechos básicos, a pesar de los avances en los indicadores económicos y sociales de la región^(14)^.

Las condiciones de salud de los residentes están profundamente influenciadas por factores socioeconómicos y culturales como la clase social, el género, el color, los ingresos, el saneamiento y el acceso a los servicios. Estos determinantes sociales afectan directamente a la adherencia a la vacunación, y la baja escolarización y los bajos ingresos se asocian a menudo con la falta de conocimientos sobre las vacunas. La indecisión ante las vacunas es menor entre las personas con mayor nivel de escolarización, mientras que las experiencias negativas con la recepción, los problemas estructurales, las creencias religiosas y las preocupaciones por la seguridad alejan a la población. Reforzar la acogida en los servicios sanitarios es una estrategia esencial para fomentar la adherencia y promover el autocuidado^(15)^.

El acto de acoger requiere algo más que poner los servicios a disposición, ya que exige la práctica de la escucha sensible, que trasciende el simple acto de escuchar. La escucha sensible se basa en la empatía, reconociendo la libertad y el valor de cada persona sin juzgarla ni compararla. Este abordaje holístico busca comprender al otro como un ser humano integral, promoviendo un vínculo de confianza que puede transformar la relación entre los profesionales de salud y la comunidad, facilitando el acceso a las vacunas y fortaleciendo la relación entre cuidado y autonomía^(16)^.

Teniendo esto en cuenta, los equipos de Atención Primaria de Salud (APS) han integrado las funciones de vigilancia en zonas urbanas, rurales y remotas, utilizando modelos como los sistemas de agregación de datos, los cuadros de mando y la vigilancia digital, que han desempeñado un papel importante durante el COVID-19. Estrategias como la vigilancia epidemiológica activa y la búsqueda activa se han visto reforzadas por la integración de las tecnologías de la información y cuidado en salud (TICS), como el uso de las redes sociales, incluido *Instagram* ^®^, para difundir información sobre la vacunación. Estas prácticas amplían el alcance de las acciones de salud al facilitar la producción, el almacenamiento, la transmisión y el acceso seguro a la información, lo que demuestra la importancia de la innovación tecnológica en la promoción de la salud^(17)^.

En Brasil, la política de información del SUS, intensificada en la década de 2000, consolidó el uso de las TICS para mantener sistemas de información articulados y actualizados. En la APS, considerada puerta de entrada y coordinadora de la atención en la Red Asistencial, estas tecnologías han transformado el proceso de trabajo, permitiendo mayor eficacia en la llegada a los usuarios, mejoras en la enseñanza y el aprendizaje y decisiones clínicas más precisas. Además, las TICS han facilitado la construcción de diagnósticos colectivos de los territorios, fortaleciendo la capacidad de respuesta de los servicios de salud^(18)^.

La presencia de las nuevas tecnologías es una realidad y ha sido utilizada como herramienta en el proceso de síntesis, difusión y aplicación del conocimiento, como la Telesalud, la Red Universitaria de Telemedicina y la Universidad Abierta del Sistema Único de Salud. La aplicación práctica del conocimiento promueve mejoras directas en la salud de los usuarios, favoreciendo la provisión de productos y servicios más eficaces y fortaleciendo el sistema sanitario. Entre las aplicaciones más utilizadas para dispositivos móviles se encuentran *Telegram* ^®^ *, Kakaotalk* ^®^ y WhatsApp^®(19)^.

Destaca *WhatsApp* ^®^, una plataforma ampliamente utilizada en todo el mundo para mensajería instantánea que permite compartir diferentes tipos de medios, incluyendo textos, imágenes, vídeos, documentos, localización y llamadas de voz. Este recurso ofrece un acceso rápido y sencillo a una gran variedad de información, incluida la relacionada con la salud^(19)^.

Durante la pandemia, el uso de la app en APS fue una estrategia que instituyó nuevas formas de gestión de los procesos de trabajo y de atención a la salud, desde una perspectiva comunicativa, activa y sincrónica frente a los problemas identificados en los escenarios de atención, como la necesidad de diseminar información calificada, en un contexto en que se difundían *fake news* o informaciones no veraces^(20)^. Representan una preocupación relevante en la asistencia sanitaria, especialmente debido a la difusión de información falsa sobre los cuidados, que se ha intensificado durante la pandemia COVID-19.

Durante la pandemia, la desinformación ha puesto en peligro la atención domiciliaria e institucional, poniendo en riesgo vidas al comprometer la adherencia a las medidas de salud pública. En respuesta, en 2018 el Ministerio de Salud implementó un espacio en su sitio web y redes sociales dedicado a aclarar información basada en evidencia científica, buscando combatir los impactos negativos de las apps de mensajería en la difusión de rumores sobre enfermedades como fiebre amarilla, gripe y sarampión. La difusión de información falsa ha agravado aún más el descenso de los indicadores de vacunación, alejando a los usuarios de las unidades sanitarias y dificultando el control epidemiológico.

Incluso antes de la pandemia, los estudios ministeriales ya indicaban una reducción de la aceptación de vacunas entre los niños, un problema que se ha agravado y que ha provocado dudas sobre la vacunación relacionadas con la falta de conocimientos sobre las vacunas, el miedo a los efectos adversos y secundarios y la infravaloración de la letalidad de las enfermedades prevenibles mediante vacunación, acciones motivadas por las noticias falsas *(fake news)* ^(21)^. En Malasia, el acceso a los medios de comunicación social se relacionó con la indecisión sobre las vacunas, con un aumento del número de madres que tenían en cuenta las opiniones de terceros, Internet y que tenían dudas sobre la eficacia de la vacuna^(22)^.

Estudios brasileños han demostrado que el escaso conocimiento de la vacunación por parte de los padres y familiares contribuye a un programa de vacunación incompleto. Sin embargo, la acción profesional para difundir información mediante la alfabetización en salud tiene el potencial de aumentar la adherencia y el conocimiento sobre la prevención de enfermedades^(23,24)^.

Aunque otros factores también contribuyen al comportamiento inadecuado, es necesario tener en cuenta a las personas que no tienen un acceso justo y equitativo a la asistencia sanitaria, como defiende el SUS. El intercambio de *fake news* se identifica como una de las principales razones de la no aceptación de las medidas preventivas y los cuidados recomendados por la ciencia en favor de la salud en todo el mundo^(25)^.

Con base en las prácticas establecidas por el equipo de salud en este territorio, ha sido posible mejorar la cobertura de vacunación, mitigando la indecisión y la reducción de la cobertura de vacunación, que han sido problemas graves desde que se propagaron los movimientos antivacunas durante la pandemia de coronavirus^(26)^.

En Brasil, otras estrategias también han sido destacadas en la literatura reciente. Un estudio publicado en 2024 analizó iniciativas para aumentar la cobertura de vacunación en la APS de una ciudad de Pará. La investigación demostró que el éxito en el logro de la cobertura de vacunación se atribuyó a la capacitación de los equipos, especialmente de los TCS. Esta formación tenía como objetivo proporcionar información técnica para sensibilizar a las familias sobre la importancia de la vacunación. Este enfoque permitió a los profesionales sanitarios adquirir las habilidades necesarias para acercarse a las familias de forma eficaz, aclarando dudas y disipando mitos sobre las vacunas^(27)^.

Otro punto destacado por el estudio fue la búsqueda activa de personas que previamente habían sido identificadas como ausentes en los servicios de salud. Esta estrategia personalizada resultó eficaz para llegar a las personas que no acudían a las campañas de vacunación, lo que demuestra el compromiso del equipo sanitario para garantizar el acceso a las vacunas recomendadas. Además, hacer que la vacunación estuviera disponible fuera del horario de oficina fue otra medida importante, teniendo en cuenta las dificultades a las que se enfrentan algunas personas para acceder a los servicios durante el horario laboral habitual^(27)^.

Del mismo modo, un estudio realizado en Minas Gerais informó de la importancia de estrategias específicas para aumentar la cobertura de vacunación. El estudio describió talleres organizados en cuatro etapas: momento motivacional; núcleo contextual; núcleo de integración/planificación; y núcleo de integración/resultados. Estos talleres no sólo pusieron de manifiesto las realidades locales y la necesidad de mejorar los procesos de inmunización, sino que también promovieron el desarrollo de estrategias eficaces. Como resultado, hubo un aumento de la cobertura de vacunación, lo que demuestra la eficacia de la metodología utilizada^(28)^.

Los resultados de 51 experiencias de todas las regiones brasileñas sobre el tema de la inmunización - 19 del Sudeste, 14 del Sur, 12 del Nordeste, dos del Norte y cuatro del Centro-Oeste - muestran que la baja cobertura de vacunación es un gran reto al que se enfrenta la atención en salud en todo Brasil. La orientación colectiva, la vacunación fuera del horario de atención, la ampliación del horario de atención y la formación de los profesionales de la salud fueron identificadas con mayor frecuencia en las experiencias exitosas de vacunación^(29)^.

La evaluación de las repercusiones de la intervención ministerial denominada «Plan de Acción: Estrategia de Vacunación en la Frontera» y las estrategias de vinculación de los usuarios a través de la educación en salud y la adaptación de los flujos para promover el acceso se identificaron en los municipios de éxito en términos de mejora de los indicadores de vacunación.

## CONCLUSIÓN

El informe presentado mostró que las prácticas utilizadas por la ESF desempeñaron un papel fundamental en el aumento de la cobertura de vacunación (en el territorio) en la favela Vigário Geral, destacándose como modelo de excelencia en el cumplimiento de las metas de vacunación, especialmente en niños de hasta 2 años.

Las favelas de Río de Janeiro, por su vulnerabilidad, exigen una responsabilidad epidemiológica cualificada. Las intervenciones bien planificadas y ejecutadas, combinadas con el uso estratégico de las nuevas tecnologías, y la colaboración entre los profesionales de la salud y la comunidad han demostrado ser eficaces para revertir la caída de la inmunización.

Ante el reto que suponen las *fake news* para la salud pública, esta experiencia pone de relieve la importancia de contar con estrategias sólidas de comunicación y educación sanitaria para garantizar el cumplimiento de las medidas preventivas y la confianza de los usuarios en los servicios de salud. Informes como este pueden ayudar a replicar estrategias exitosas y así promover la salud y proteger contra enfermedades prevenibles por vacunación.

## Data Availability

No aplicable. Este estudio se basa en un informe de experiencia y no generó datos disponibles para compartir.
